# Precision Biomarker Identification in Gynecological Cancers Using Coexpression Networks and Attention-Based LSTM in Healthcare 4.0

**DOI:** 10.3390/diagnostics16040546

**Published:** 2026-02-12

**Authors:** Sakib Sarker, Emon Ahammed, Md. Faruk Hosen, Mohammad Badrul Alam Miah, Mohammad Amanul Islam, Deepak Ghimire, Youngbae Hwang, A. S. M. Sanwar Hosen

**Affiliations:** 1 Department of Computer Science and Engineering, Uttara University, Dhaka 1230, Bangladesh; sakib.sarker@uttarauniversity.edu.bd (S.S.); amanul.islam@uttarauniversity.edu.bd (M.A.I.); 2 Department of Computer Science and Engineering, Mymensingh Engineering College, Mymensingh 2208, Bangladesh; emonahammed1999@gmail.com; 3 Department of Computer Science and Engineering, Netrokona University, Netrokona 2400, Bangladesh; farukictmbstu@gmail.com; 4 Department of ICT, Mawlana Bhashani Science and Technology University, Tangail 1902, Bangladesh; badrul.ict@mbstu.ac.bd; 5 IT Application Research Center, Korea Electronics Technology Institute, Jeonju 54853, Republic of Korea; deepak@keti.re.kr; 6 Department of Intelligent Systems & Robotics, Chungbuk National University, Cheongju 28644, Republic of Korea; 7 Department of Artificial Intelligence and Big Data, Woosong University, Daejeon 34606, Republic of Korea

**Keywords:** biomarkers, WGCNA, machine learning, bioinformatics, hub genes, attention-based LSTM

## Abstract

**Background:** Cervical cancer (CC) and ovarian cancer (OC) are among the most prevalent and lethal gynecological malignancies in women, necessitating the identification of reliable biomarkers for early diagnosis and prognosis. **Methods:** This study integrates bioinformatics and Healthcare 4.0 to identify key biomarkers associated with these cancers. Differentially expressed genes (DEGs) were identified from two microarray datasets. mRMR followed by SVM-RFE was applied to the identified DEGs to extract the most significant ML-based DEGs (MDEGs). The predictive ability of the selected gene subsets was further evaluated via multiple classifiers, where attention-based long short-term memory (AttLSTM) consistently achieved the best performance across both datasets. In parallel, WGCNA was conducted to identify coexpression-associated genes (CAGs) from significant modules in each dataset. A PPI network (PPIN) was constructed using the genes common to MDEGs and CAGs and was analyzed via Cytoscape. **Results:** Four hub genes, MCM3, FOXM1, SH3BP5, and PAPSS2, were identified via the degree method. mRNA expression analysis revealed that FOXM1 and MCM3 were upregulated, whereas SH3BP5 and PAPSS2 were downregulated in cancer tissues compared with normal tissues. ROC curve analysis demonstrated the high prognostic significance of these hub genes, with substantial AUC scores indicating strong discriminatory power. Furthermore, molecular docking analysis with an FDA-approved drug compound confirmed the significant binding affinity between these genes and the drug molecules. **Conclusions:** These findings suggest that FOXM1, MCM3, SH3BP5, and PAPSS2 could serve as biomarkers for early prognosis, diagnosis, and targeted therapy in patients with cervical and ovarian cancer.

## 1. Introduction

Cervical cancer (CC) ranks as the fourth most prevalent malignancy affecting women globally [1] and remains one of the most prevalent female malignancies. Its development is mainly driven by persistent infection with perilous human papillomavirus (HPV) [2]. An estimated 600,000 new cases and 342,000 deaths occurred worldwide in 2020 [3]. While its incidence has decreased in the United States, it remains a leading cause of cancer-related mortality in developing countries [1]. Ovarian cancer (OC) is one of the most frequently diagnosed gynecological cancers and remains a leading cause of death related to gynecological malignancies worldwide. In 2018, approximately 250,000 new cases and 160,000 deaths were reported [4]. Several risk parameters are linked with OC, including a family medical record of breast cancer or OC, obesity, smoking, early menstruation, late menopause, and not giving birth. Because of its subtle manifestation, more than 70% of OC cases are diagnosed in later stages [5]. Both cervical and ovarian cancers affect the female reproductive system and are associated with high mortality rates [6]. Owing to the high mortality associated with these cancers, biomarkers can serve an indispensable role in their early diagnosis and prognosis.

Recently, gene expression analysis has become a key approach for identifying important genes associated with various cancers. Yang et al. [7] identified six HGs (IGFBP5, KRT1, IVL, CCNA2, AURKA, and TOP2A) through a protein–protein interaction (PPI) network. They reported upregulated expression of CCNA2, AURKA, and TOP2A, along with downregulated expression of IGFBP5 in cervical cancer samples. Karunakara et al. [8] conducted an in-depth analysis of the miR-497/195 cluster to assess its prognostic value and role in CC. They identified ten HGs from a PPIN and found that the upregulated expression of three genes (RECK, ATD5, and BCL2) and the downregulated expression of HIST1H3H, RCAN3, and OSBPL3 influenced the overall survival of CC patients. Honar et al. [9] performed a comprehensive analysis of high-grade primary tumors (HGPTs) and identified the top ten HGs from their PPIN. They also identified key transcription factors (FOXM1, NFYA, TCF7L2, VDR, and SIN3A) associated with HGPTs. Additionally, they highlighted NUSAP1, CDCA5, CKS2, PRC1, and CEP55 as potential biomarkers. Shihao et al. [10] implemented a coexpression network analysis alongside differential expression analysis. They identified three HGs (CLDN3, IRF6, and PRSS8) from the PPI network and reported that IRF6 was strongly associated with OC development and progression.

Although cervical and ovarian cancers have been widely studied, there are still opportunities for further research. Most previous studies have focused primarily on identifying hub genes within PPI networks. However, discovering essential genes or reliable biomarkers remains a significant challenge in genetic data analysis. The challenge arises from the sparse nature of high-dimensional gene expression data, which complicates effective analysis and leads to the phenomenon known as the “curse of dimensionality” [11]. Additionally, these datasets often contain redundant information, since genes with identical expression patterns introduce redundancy [12]. Recent developments in Healthcare 4.0, the fourth revolution in healthcare, offer transformative potential for addressing these challenges. Healthcare 4.0 integrates pioneering technologies such as artificial intelligence (AI), big data analytics, the Internet of Things (IoT), cloud computing, and smart interconnected medical devices to enable real-time personalized healthcare [13]. By leveraging Healthcare 4.0 principles, the identification of crucial gene expression biomarkers for various cancers can be significantly enhanced through sophisticated machine learning and bioinformatics techniques. Utilizing these opportunities, integrated machine learning (ML) and bioinformatics approaches [14,15,16] have gained increasing adoption in recent years. Among these methods, the effectiveness of the mRMR method lies in its ability to precisely select the most relevant genes linked to disease while effectively reducing redundancy among genes [12,17]. Furthermore, SVM-RFE enhances feature selection by identifying the most insightful subset of genes, improving the robustness of biomarker discovery [18].

**Figure 1:**
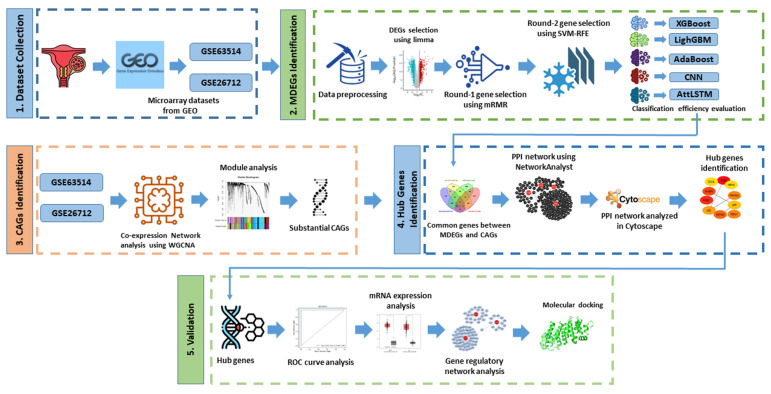
Comprehensive pipeline for identifying candidate biomarkers associated with cervical cancer and ovarian cancer.

In this study, two microarray datasets were utilized, one for each disease. The analysis was conducted from two perspectives: ML-based DEG identification and WGCNA-based gene identification. For ML-based DEGs, we first identified DEGs from both datasets, followed by a two-step gene selection process. High-dimensional gene expression data contain thousands of features but limited samples, increasing noise, redundancy, and overfitting risk. To address this, a two-step feature selection strategy combining mRMR and SVM-RFE was applied. mRMR first selects genes highly relevant to the phenotype while minimizing redundancy, and SVM-RFE further refines this set by iteratively removing less discriminative features, yielding a compact and informative gene signature considered as ML-based DEGs (MDEGs). To model complex nonlinear gene interactions, an AttLSTM network was employed. The LSTM captures feature dependencies, while the attention mechanism emphasizes the most informative genes, improving representation learning and classification robustness. Together, this framework enhances biomarker discovery by reducing dimensional noise and enabling more effective learning of gene interaction patterns. Simultaneously, coexpression networks were constructed via WGCNA, and significant coexpression-associated genes (CAGs) were extracted from key modules. Functional annotation was performed using clusterProfiler. A PPIN was then constructed using the genes common to MDEGs and CAGs. The identified hub genes (HGs) were further validated by evaluating their discriminative power through receiver operating characteristic (ROC) curves and analyzing their mRNA expression levels in tumor samples. Additionally, we conducted comprehensive gene regulatory analyses as well as molecular docking to analyze the binding affinity of the HGs and FDA-approved drug compound for the treatment of cervical and ovarian cancer. The complete framework is presented in Figure 1 and system Algorithm 1.
**Algorithm 1:** Proposed Bioinformatics and Machine Learning-Based Biomarker Identification Framework**Input:** Microarray datasets for cervical and ovarian cancer.**Output:** Identified key candidate biomarkers and their drug–target interaction profiles.**1: Differential Expression Analysis:****2:**     Identify DEGs using the limma package.**3: Two-Step Gene Selection via Machine Learning:****4:**     **Step 2.1: mRMR-based Gene Subset Selection****5:**        Apply mRMR to minimize redundancy and maximize relevance among DEGs.**6:**        Select optimal gene subset α′.**7:**     **Step 2.2: Gene Subset Refinement Using SVM-RFE****8:**        Train an SVM classifier on α′ with class labels.**9:**        Compute feature ranking weights via recursive feature elimination (RFE).**10:**        Iteratively remove least significant genes.**11:**        Obtain final optimal gene subset α (MDEGs).**12: Co-Expression Network Construction via WGCNA:****13:**     Build co-expression network using WGCNA.**14:**     Identify gene modules significantly linked with cancer phenotypes.**15:**     Extract module genes as Co-Expression-Associated Genes (CAGs).**16:**     Identify intersecting genes between MDEGs and CAGs.**17: Functional Enrichment and PPIN Analysis:****18:**     Perform GO and KEGG enrichment using clusterProfiler.**19:**     Construct PPI network via NetworkAnalyst and analyze in Cytoscape.**20:**     Identify hub genes (HGs) via degree centrality.**21: Validation of Identified Hub Genes:****22:**     Evaluate discriminative power through ROC analysis.**23:**     Analyze expression patterns using GEPIA.**24:**     Perform gene regulatory network analysis using NetworkAnalyst.**25: Molecular Docking:****26:**     Perform molecular docking with an FDA-approved drug.

## 2. Materials and Methods

### 2.1. Background Study

In biomarker discovery research, many earlier studies primarily relied on conventional bioinformatics and systems biology approaches. Although machine learning methods have recently gained popularity, most investigations employ shallow feature selection techniques that may overlook highly informative genes. In contrast, the present study implements a distinct two-step gene selection strategy to enhance discriminative gene prioritization, followed by an attention-based LSTM model to capture complex, nonlinear relationships among gene features. Additionally, coexpression analysis was incorporated to identify biologically relevant gene modules. A detailed comparison with previous studies is presented in Table 1.

### 2.2. Microarray Data Acquisition

Two gene expression microarray datasets were curated from the publicly available Gene Expression Omnibus (GEO) database. The GSE63514 [22] dataset was collected for cervical cancer, whereas the GSE26712 [23,24] dataset was collected for ovarian cancer. The GSE26712 dataset is based on the GPL96 platform, while the GSE63514 dataset is founded on the GPL570 platform. Additional details about these datasets are outlined in Table 2.

### 2.3. Data Preprocessing and Screening of DEGs

Differential expression (DE) analysis is a molecular biology technique used to uncover genes with significantly altered expression levels among various sample groups, such as healthy and diseased tissues. The primary aim of DE is to pinpoint genes linked to specific biological conditions, helping to reveal underlying mechanisms, disease associations, and potential responses to treatment by providing insights into gene regulation and related biological processes [25]. Batch effects arising from differences in experimental conditions, platforms, or sample processing can introduce non-biological variation that may confound downstream analyses. To minimize such technical bias and improve data comparability, batch correction was performed using the ComBat function implemented in the sva toolkit in R. This criterion applies an empirical Bayes approach to adjust for systematic batch-related variation while preserving true biological differences between sample groups [26]. The datasets underwent $log_{2}$ transformation and quantile normalization before DE analysis was conducted. We conducted DE analysis via the limma package [27] in R to compare the expression values among normal and tumor samples and identify DEGs. In cases where several probes corresponded to a single gene symbol, the probe exhibiting the lowest adjusted *p*-value was designated to represent the gene in the analysis. A set of DEGs, $\alpha^{\prime \prime }$ was created by identifying DEGs using the cut-off: *adj.p-value* < 0.05 and |logFC|>1.

### 2.4. ML-Based Gene Subset Selection

In the era of Healthcare 4.0 [28], utilizing emerging analytical approaches such as artificial intelligence and big data analytics may enable precision diagnosis through the integration of high-dimensional genomic data. However, the vast volume and complexity of gene expression datasets pose significant challenges, which impede accurate biomarker identification. Feature selection is a critical step to enhance the interpretability and predictive power of models by isolating the most relevant and non-redundant genes. Efficient feature selection methods contribute directly to the discovery of reliable biomarkers, facilitating early diagnosis and personalized treatment strategies in line with the goals of Healthcare 4.0 [17].

#### 2.4.1. mRMR-Based Gene Subset Selection

Genomic expression datasets contain thousands of genes, leading to the curse of dimensionality. ML-based feature selection helps reduce this complexity by identifying the most significant genes [29]. Empirical Bayes-moderated tests with FDR adjustments are commonly used to identify DEGs. However, this approach does not eliminate redundancy among pinpointed genes. To address this, the mRMR approach selects features by minimizing redundancy and maximizing relevance, improving gene subset quality [30]. As a highly robust feature selection technique in ML, the mRMR algorithm has gained widespread application in multi-omics study in recent times [12].

In Round-1 of gene subset selection, mRMR was applied to the identified DEGs ($\alpha^{\prime \prime }$) in the respective datasets. This method was applied to select an optimal subset of DEGs that balances high relevance to the cancer phenotype with low redundancy among themselves. The mathematical representation for the mRMR technique is as follows [12,31,32]:

Relevance of each gene $g_{i}$ to the target variable *c* (cancer status) was quantified via mutual information:

(1) $$\mathrm{Relevance}\left(g_{i},c\right)=I\left(g_{i};c\right)$$

where $I(g_{i};c)$ denotes the mutual information between gene $g_{i}$ and class label *c*.

Redundancy between pairs of genes $g_{i}$ and $g_{j}$ was measured similarly by mutual information:

(2) $$\mathrm{Redundancy}\left(g_{i},g_{j}\right)=I\left(g_{i};g_{j}\right)$$

mRMR optimizes the selection of a gene subset *S* by maximizing the difference between average relevance and average redundancy:

(3) $$\max_{S}\left[\frac{1}{\left|S\right|}\sum_{g_{i}\in S}I\left(g_{i};c\right)-\frac{1}{{\left|S\right|}^{2}}\sum_{g_{i},g_{j}\in S}I\left(g_{i};g_{j}\right)\right]$$

This criterion ensures the chosen genes collectively provide maximal information about the phenotype while minimizing overlapping information among them. The genes were ranked according to their mRMR scores, and the gene subset $\alpha^{\prime }$, with the top-ranked genes was passed to the subsequent SVM-RFE step.

#### 2.4.2. Gene Subset Creation via SVM-RFE

The support vector machine (SVM) classifier is a supervised machine learning method that aims to find an optimal hyperplane in a high-dimensional space to maximize the margin between classes [33]. The SVM classifier separates two classes by finding a hyperplane f(x), defined as [33,34]:

(4) $$f\left(x\right)=\mathrm{sign}\left(\sum_{i=1}^{n}\alpha_{i}y_{i}K\left(x_{i},x\right)+b\right)$$

where *x* is the input feature vector, $\alpha_{i}$ is the Lagrange multiplier, $y_{i}$ is the class label (+1 or −1), $K(x_{i},x)$ is the kernel function (linear, polynomial, Gaussian, etc.), and *b* is the bias term.

Support vector machine-recursive feature elimination (SVM-RFE) [34,35] was applied to the gene subset $\alpha^{\prime }$ to identify a new, more significant gene subset α, which was classified as MDEGs. To prevent information leakage, SVM-RFE–based gene subset selection was performed within a fully nested stratified cross-validation framework. For each training fold, mRMR ranking followed by SVM-RFE was applied exclusively to the training data, and the resulting gene subset was then used to evaluate classification performance on the corresponding unseen test fold. The final gene subset α, obtained from this two-step feature selection, was then used as input for multiple classifiers, including XGBoost, convolutional neural network (CNN), AdaBoost, LightGBM, and attention-based long short-term memory (AttLSTM), to evaluate classification performance. To benchmark the effectiveness of the proposed two-step strategy, it was compared against single-step feature selection algorithms, including LASSO, ANOVA, mutual information gain (MiG), and extra trees.

### 2.5. WGCNA and Module Analysis

Weighted gene coexpression network analysis (WGCNA) is a systems biology approach that analyzes gene expression data to identify patterns of correlation among genes across microarray or RNA-seq samples. It builds a gene coexpression network, detects modules of closely related genes, and links these modules to clinical traits or external characteristics. The main objective of WGCNA is to discover biologically relevant gene groups and potential hub genes that could act as biomarkers or therapeutic targets [36]. Both of our microarray datasets (GSE26712 and GSE63514) were utilized for our analysis and performed coexpression analysis via the WGCNA library in R. Initially, phenotypic data for the study samples were retrieved from the NCBI GEO database using the GEOquery package. To ensure data quality, we used the goodSamplesGenes() function from WGCNA to screen out genes and samples with excessive missing values or outliers. The outlier samples were further detected through hierarchical clustering via the hclust() function and were excluded to prevent distortion of the analysis.

Next, the pickSoftThreshold() procedure was utilized to derive the efficient soft-thresholding power (β) for constructing the adjacency matrix. Soft-thresholding powers ranging from 1 to 50 (incrementing by 1 initially, then by 2 from 12 onward) were tested. The optimal power was selected on the basis of the criterion $R^{2}\geq 0.80$. Using the selected soft-thresholding power, we constructed the adjacency matrix and converted it into a topological overlap matrix (TOM). The TOM dissimilarity measure (1 − TOM) was computed, followed by hierarchical clustering with the average linkage method to cluster genes with concordant expression profiles into distinct coexpression modules. Each module was assigned a unique color. Then, gene co-expression architecture was inferred via the blockwiseModules() method, setting the following parameters: power = best threshold power, minModuleSize = 30, deepSplit = 1, TOMType = “unsigned”, mergeCutHeight = 0.25, and randomSeed = 1234. To visualize the clustering results, we generated dendrograms with module colors before and after merging via the plotDendroAndColors() function.

We then computed module eigengenes (MEs) to reflect the overall measure of expression profiles of genes within each module. These eigengenes were used to assess module-trait relationships, where we analyzed the associations between MEs and clinical traits (healthy controls vs. cases). The strength of these associations was visualized in a heatmap, with correlation coefficients indicating their significance. Finally, significant coexpression-associated genes (CAGs) were identified from key modules for subsequent investigations.

### 2.6. Pathway Enrichment Analysis

This analysis delineates biological pathways significantly correlated with genes exhibiting differential expression or segregated sample clusters. It supports biomarker discovery by highlighting genes that are overrepresented in critical pathways and aids validation by uncovering their functional roles and interactions at the protein level [25]. We performed Gene Ontology (GO) [37] and Kyoto Encyclopedia of Genes and Genomes (KEGG) [38] pathway enrichment analyses on the common MDEGs and CAGs between the two datasets individually via the clusterProfiler [39] package in R and ClueGO [40]. The GO annotations were stratified into three distinct functional classes: biological process (BioProc), cellular component (CellCom), and molecular function (MoleFunc). A relevance threshold of p<0.05 was used to identify statistically notable enrichments.

### 2.7. PPIN Construction and HG Identification

A PPIN offers a systematic framework for uncovering disease-associated genes by assessing interactions between functionally related genes [41]. Common genes between MDEGs and CAGs were identified, and a PPIN was constructed using NetworkAnalyst. The network was then visualized and examined in Cytoscape v3.0. HGs were recognized by applying the degree centrality in the cytoHubba [42] feature, genes with at least 10 interactions were selected.

### 2.8. Validation of the Identified HGs

#### 2.8.1. Diagnostic Efficacy Evaluation via ROC Analysis

For the validation of the identified HGs, their diagnostic potential in distinguishing normal and cancer samples was assessed. To evaluate their discriminative power, we applied leave-one-out cross-validation (LOOCV) protocol with logistic regression (LR) on both the GSE63514 and GSE26712 datasets. Logistic regression, a widely used method for binary classification, was chosen beacuse of its effectiveness in modeling the relationship between gene expression levels and disease status [43]. In addition, the diagnostic performance of the identified hub genes was evaluated using two independent GEO datasets (GSE9750 and GSE37582).

#### 2.8.2. mRNA Expression Levels and Survival Analysis

GEPIA serves as an online interactive interface designed for rapid and customizable analysis of gene expression data derived from TCGA and GTEx cohorts [44]. To validate the identified HGs, GEPIA was employed to interpret their expression levels in CC and OC tissues relative to adjacent healthy control samples. The analysis was visualized using box plots, with expression values represented as log2(TPM + 1). Significant differential expression was determined using the criteria |logFC|>1 and p<0.01. To validate the prognostic efficiency of the unveiled HGs and to examine their association with patient outcomes, survival analysis was performed using the TCGA-CESC dataset. The prognostic efficacy of each hub gene was evaluated by stratifying CESC patients into high and low-risk groups on the basis of median gene expression level.

### 2.9. Gene Regulatory Networks Analysis

Gene regulatory networks (GRNs) define the regulatory relationships between genes, providing insights into their biological roles, including molecular functions and broader functional activities [45]. In this study, we analyzed two types of GRNs: transcription factor (TF)–gene (TFG) interactions and gene–miRNA interactions. TFG interactions illustrate how genes collaborate within regulatory networks and pathways [46]. To analyze TF–gene interactions for the common genes, we used NetworkAnalyst, leveraging the ENCODE database integrated within the platform to establish the TFG network. Similarly, gene—miRNA interactions were also analyzed via NetworkAnalyst.

### 2.10. Molecular Docking Analysis

Molecular docking (MolDock) was conducted to evaluate the couplings between potential biomarkers and drug commonly used in cervical and ovarian cancer treatment. The 3D frameworks of the corresponding proteins were obtained from the Protein Data Bank (PDB). The molecular structures of proteins were formulated using AutoDock Tools (version 1.5.7) by removing water molecules, incorporating polar hydrogens, and converting them into PDBQT format for docking. For the drug molecule, we retrieved the 3D structures of the Olaparib drug compounds (collected from NCBI) from PubChem in SDF format. The molecules were visualized and adapted into the appropriate configuration using PyMOL (version 3.1.3.1). The binding interactions between the drug and the target proteins were analyzed using MolDock, with visualization performed in PyMOL to reveal critical insights into their potential interactions.

## 3. Results

### 3.1. Screening of DEGs

**Figure 2:**
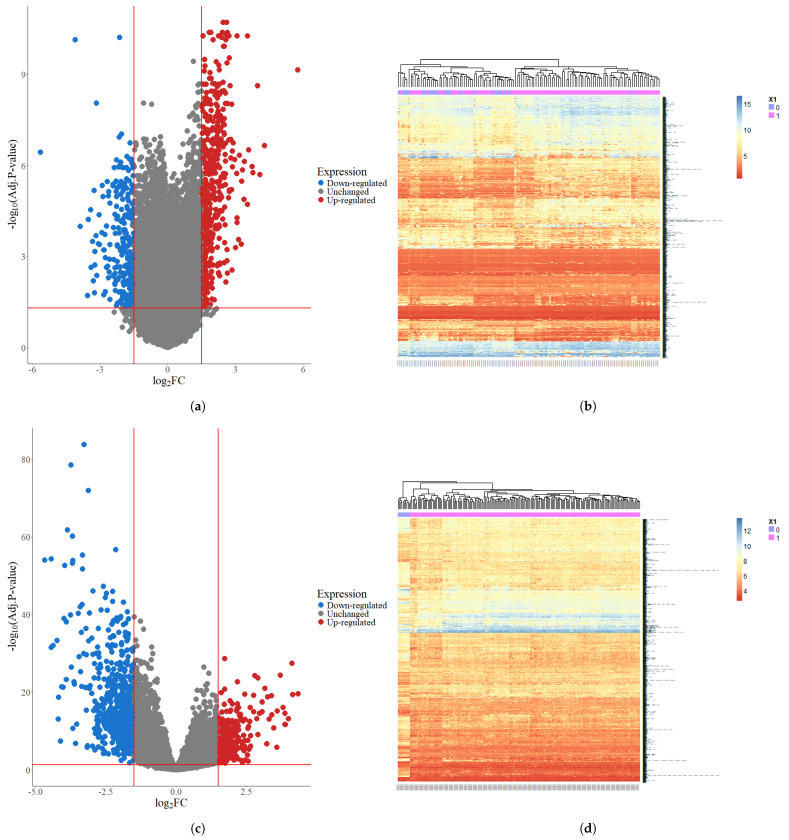
Volcano plots and heatmaps representing DEGs in the microarray datasets for cervical and ovarian cancer. (**a**) Volcano plot of DEGs in GSE63514, where red and blue dots indicate significantly upregulated and downregulated genes. (**b**) Heatmap of DEGs in GSE63514, showing hierarchical clustering of gene expression patterns. (**c**) Volcano plot of DEGs in GSE26712, highlighting upregulated and downregulated genes. (**d**) Heatmap of DEGs in GSE26712, illustrating gene expression variations across samples.

Two microarray datasets were collected from the GEO with the accession IDs GSE63514 for cervical cancer and GSE26712 for ovarian cancer. DE analysis was performed using the limma package in R, applying thresholds of an *adj.p-value* < 0.05 and |logFC|>1 to identify DEGs. From the GSE63514 dataset, a total of 1699 DEGs were identified, including 1122 upregulated (upDEGs) and 577 downregulated genes (downDEGs). Similarly, in the GSE26712 dataset, we identified 2013 DEGs, comprising 723 upDEGs and 1290 downDEGs. The corresponding volcano plots and heatmaps illustrating these results are presented in Figure 2.

### 3.2. Two-Step Gene Selection

In Round 1 of our two-step gene selection approach, mRMR was employed as the primary feature selection method. This step aimed to reduce redundancy by eliminating genes with similar expression patterns while retaining those most relevant to the sample types. By applying mRMR, the top 1200 DEGs from both datasets were selected, ensuring a refined gene subset for Round 2.

In the second round, SVM-RFE was implemented to further optimize the gene subset obtained from Round 1. Through this process, we selected the most substantial gene subset with the top 1000 genes, and refined the feature set for improved classification performance. These genes were considered ML-based DEGs (MDEGs).

As shown in Table 3, the classifiers trained on the gene subsets obtained through two-step feature selection (mRMR+SVM-RFE) achieved strong predictive performance on both datasets. For the cervical cancer dataset (GSE63514), AttLSTM outperformed all other models with 96.15% accuracy, 0.9904 AUC, and a nearly perfect balance between precision (0.9523) and recall (1.000), followed by LightGBM and XGBoost, with accuracies above 88%. For the ovarian cancer dataset (GSE26712), the performance of all classifiers was generally greater, with accuracies exceeding 97% and AUC values close to 1.0. Notably, AttLSTM again achieves the best results, with 99.28% accuracy, 0.9982 F1, and perfect precision and recall, while CNN and XGBoost also yield strong outcomes.

Table 4 presents the comparative performance of mRMR+SVM-RFE against several single-step feature selection methods, including LASSO, ANOVA, MiG, and extra trees, on both datasets. For GSE63514, although all single-step methods achieved noticeable results with accuracies above 90%, mRMR+SVM-RFE provided the best outcome with 96.15% accuracy, 0.9904 AUC, and the highest F1 score (0.9756), alongside perfect recall (1.000). Similarly, for GSE26712, whereas ANOVA and LASSO performed strongly with accuracies of 97.48% and 96.72%, respectively, and near-perfect AUC values, mRMR+SVM-RFE again outperformed all the other methods, achieving 99.28% accuracy, 1.000 AUC, and nearly flawless balance across all the metrics (F1 score, recall, and precision all above 0.998). These results highlight the efficacy of the two-stage gene selection strategy in identifying highly discriminative biomarkers and demonstrate the superior classification ability of AttLSTM over conventional machine learning models.

### 3.3. WGCNA-Based Gene Selection

**Figure 3:**
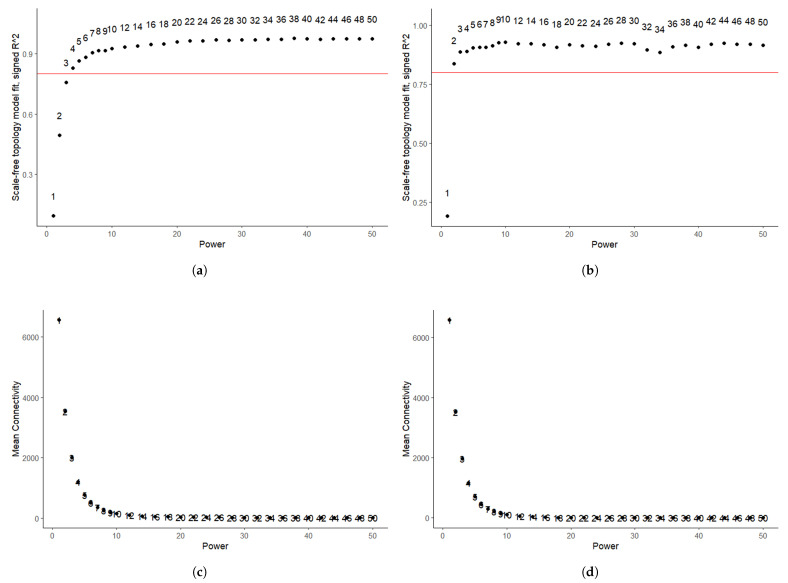
Determination of the optimal soft-thresholding power for network construction in WGCNA. (**a**,**b**) Scale-free topology model fit index (signed $R^{2}$) as a function of power for GSE63514 and GSE26712; (**c**,**d**) mean connectivity as a function of power for GSE63514 and GSE26712. The red horizontal line in (**a**,**c**) indicates the threshold for selecting an appropriate soft-thresholding power.

**Figure 4:**
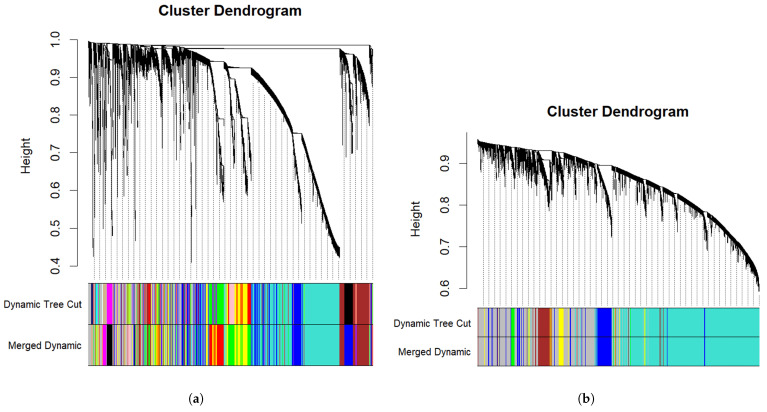
Gene coexpression module detection via hierarchical clustering. (**a**) Cluster dendrogram for the GSE63514 dataset; (**b**) cluster dendrogram for the GSE26712 dataset. In both figures, genes are grouped into modules on the basis of similarity, with the initial dynamic tree cut displayed in the first color row and the merged dynamic modules shown in the second row.

**Figure 5:**
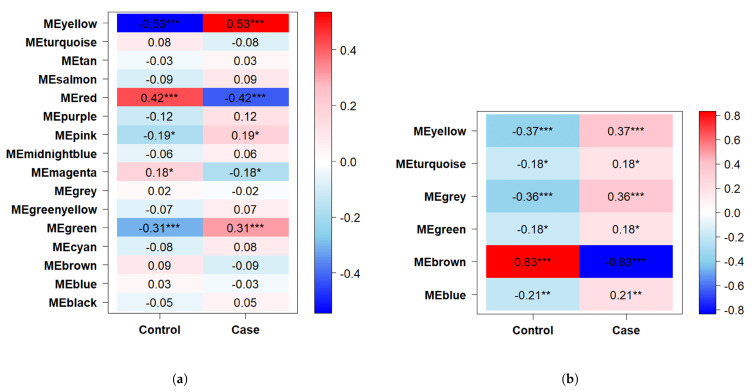
Module-trait relationship analysis. (**a**) Association between gene clusters and traits in the GSE63514 dataset; (**b**) correlation between gene coexpression modules and phenotypic traits in the GSE26712 dataset. The asterisks indicate statistical significance levels of the correlation.

This analysis was begun by identifying and removing outliers via hierarchical clustering with the hclust() function. Next, we uncovered the appropriate soft thresholding power (β) for both datasets, a critical step in constructing a scale-free network for coexpression analysis. As shown in Figure 3, the optimal soft threshold was β=4 for GSE63514 and β=2 for GSE26712. Next, the respective coexpression networks were constructed with the corresponding dendrograms presented in Figure 4. In the GSE63514 dataset, 16 modules were identified, whereas 6 distinct modules were found in the GSE26712 dataset. After analyzing the module-trait relationships shown in Figure 5, the yellow module in GSE63514 and the brown module in GSE26712 were found to be significantly associated with the trait of interest. The yellow module in GSE63514 contained 1248 genes. The brown module in GSE26712 comprised 925 genes. These genes were considered coexpression-associated genes (CAGs). We then identified the genes common to these CAGs and the MDEGs identified earlier. A total of 8 common genes were found, which were further analyzed for their potential significance.

### 3.4. Functional Enrichment Analysis

**Figure 6:**
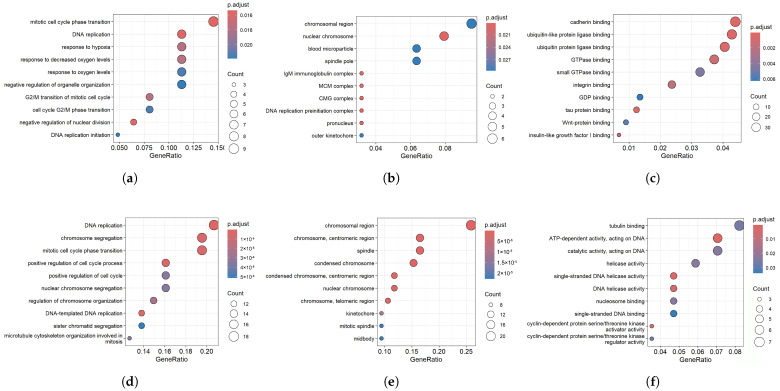
Gene ontology of the common MDEGs and CAGs: (**a**) BioProc category; (**b**) CellCom category; (**c**) MoleFunc category of the shared MDEGs. (**d**) BioProc category; (**e**) CellCom category; (**f**) MoleFunc category of the common CAGs.

Enrichment analysis was conducted separately on the common MDEGs and CAGs identified in both datasets via the clusterProfiler toolkit in R. This analysis included KEGG pathway and GO enrichment to explore the biological significance of these genes. The enrichment analysis results for MDEGs and CAGs are illustrated in Figure 6. Within the BioProc category shown in Figure 6a, the common MDEGs were significantly associated with mitotic cell cycle phase transition, DNA replication, adaptation to low oxygen conditions, the cellular response to reduced oxygen availability, and control of oxygen homeostasis. As demonstrated in Figure 6b, in the CellCom category, these genes were enriched in chromosomal regions, nuclear chromosomes, blood microparticles, spindle poles, and IgM immunoglobulin complexes. Moreover, in the MoleFunc category (Figure 6c), the key enriched terms included cadherin binding, ubiquitin-like protein ligase binding, ubiquitin protein ligase binding, GTPase binding, and small GTPase binding. In the BioProc category shown in Figure 6d, the most enriched terms for CAGs included nuclear chromosome segregation, positive regulation of the cell cycle process, mitotic cell cycle phase transition, chromosome segregation, and DNA replication. The CellCom enrichment (Figure 6e) highlighted the localization of structural components such as the chromosomal region, chromosome centromeric region, spindle, condensed chromosome, and nuclear chromosome, reinforcing their involvement in mitotic processes. With respect to MoleFunc presented in Figure 6f, the CAGs were significantly associated with helicase activity, single-stranded DNA helicase activity, ATP-dependent activity acting on DNA, catalytic activity acting on DNA, and tubulin binding.

**Figure 7:**
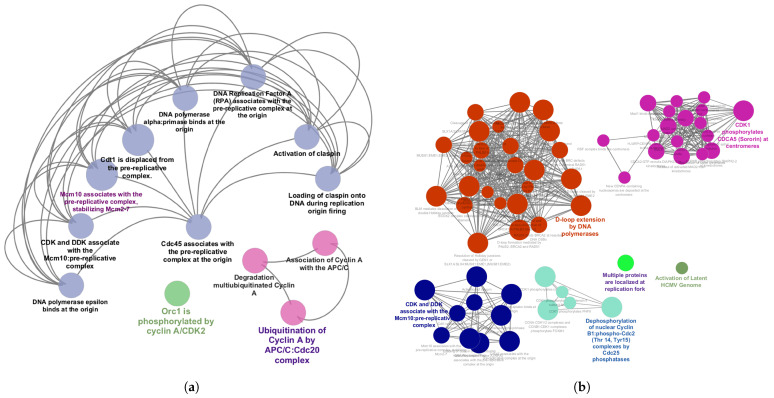
KEGG pathway mapping of overlapping (**a**) MDEGs and (**b**) CAGs. In this KEGG pathway mapping figure, the different colors represent distinct functional modules.

KEGG pathway enrichment analysis of the common MDEGs and CAGs revealed strong associations with DNA replication and cell cycle regulation. Common MDEGs (Figure 7a) were enriched mainly in DNA replication–related processes, including prereplicative complex assembly, DNA polymerase loading, Cdc45 association, and Cyclin A ubiquitination. In contrast, common CAGs (Figure 7b) were enriched in diverse biological modules, such as homologous recombination and DNA repair, cyclin/CDK-mediated cell cycle regulation, and centromere/kinetochore assembly.

### 3.5. PPIN Construction and HG Identification

**Figure 8:**
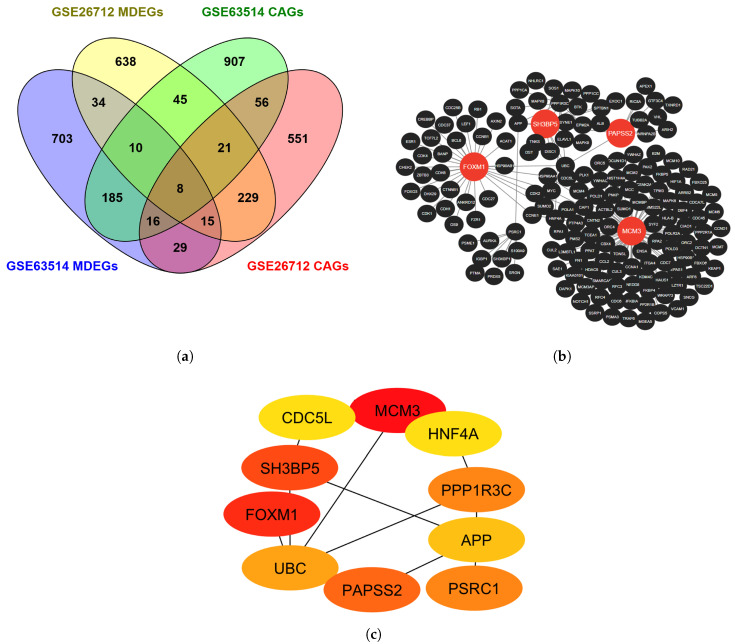
Identification and interaction analysis of genes common to MDEGs and CAGs. (**a**) Venn diagram illustrating the overlap of MDEGs and CAGs across two datasets (GSE26712 and GSE63514); (**b**) PPIN constructed for the common genes via the NetworkAnalyst database, with HGs highlighted in red; (**c**) Subnetwork of the top HGs identified using the degree method, where color intensity represents connectivity, with red indicating the highest degree of interaction.

A PPIN was constructed for the eight common genes shared between MDEGs and CAGs (presented in Figure 8a) via the NetworkAnalyst database. The network was subsequently visualized and analyzed in Cytoscape. It comprised 168 nodes and 176 edges illustrated in Figure 8b, representing the complex interactions among these genes. The top ten HGs were via the degree method in the cytoHubba feature (presented in Figure 8c). From these, genes exhibiting a minimum of ten interactions were selected for further analysis. This analysis led to the identification of four HGs—FOXM1, MCM3, SH3BP5, and PAPSS2—which exhibited the highest connectivity.

### 3.6. Validation of Identified HGs

#### 3.6.1. Diagnostic Efficacy Evaluation via ROC Analysis

**Figure 9:**
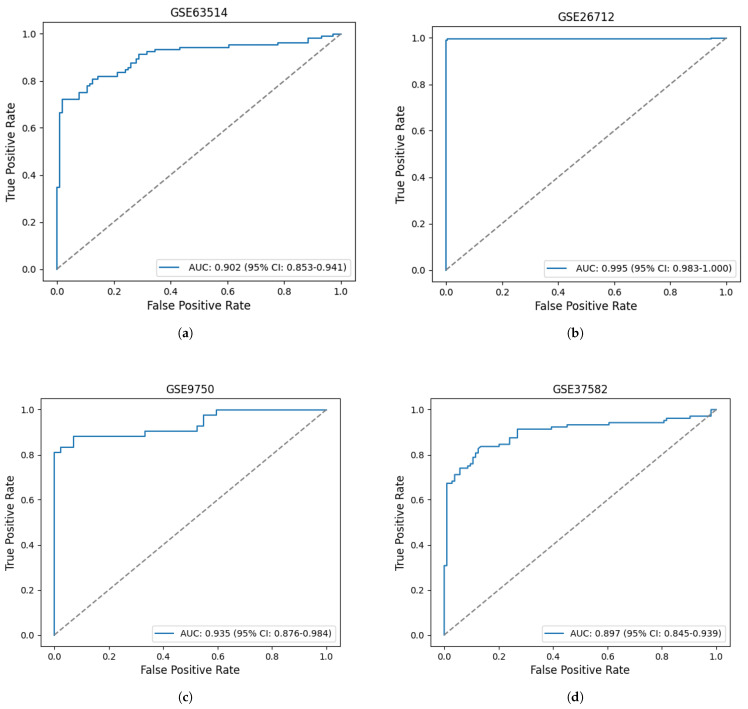
Discriminative power evaluation through ROC curve analysis in our experimental and independent test datasets. (**a**) ROC curve for the GSE63514; (**b**) ROC curve for the GSE26712 dataset; (**c**) ROC curve for the GSE9750 (independent test dataset); (**d**) ROC curve for the GSE37582 (independent test dataset). The dotted line represents the random guessing.

To validate the diagnostic capability of the identified HGs FOXM1, MCM3, SH3BP5, and PAPSS2, their diagnostic performance was assessed by calculating AUC scores from the ROC curves generated via logistic regression on both cervical and ovarian cancer datasets. As shown in Figure 9, the HGs attained an AUC of 0.902 (95% CI: 0.853–0.941) in the GSE63514 dataset. Similarly, in the GSE26712 dataset, the identified HGs attained an AUC score of 0.995 (95% CI: 0.983–1.000), indicating higher classification accuracy. In the independent test datasets, the unveiled hub genes achieved satisfactory performance, with AUC values of 0.935 (95% CI: 0.876–0.984) and 0.897 (95% CI: 0.845–0.939). These findings indicate the substantial discriminatory power of the pinpointed HGs in effectively distinguishing normal and cancer samples.

#### 3.6.2. mRNA Expression Level and Survival Analysis

**Figure 10:**
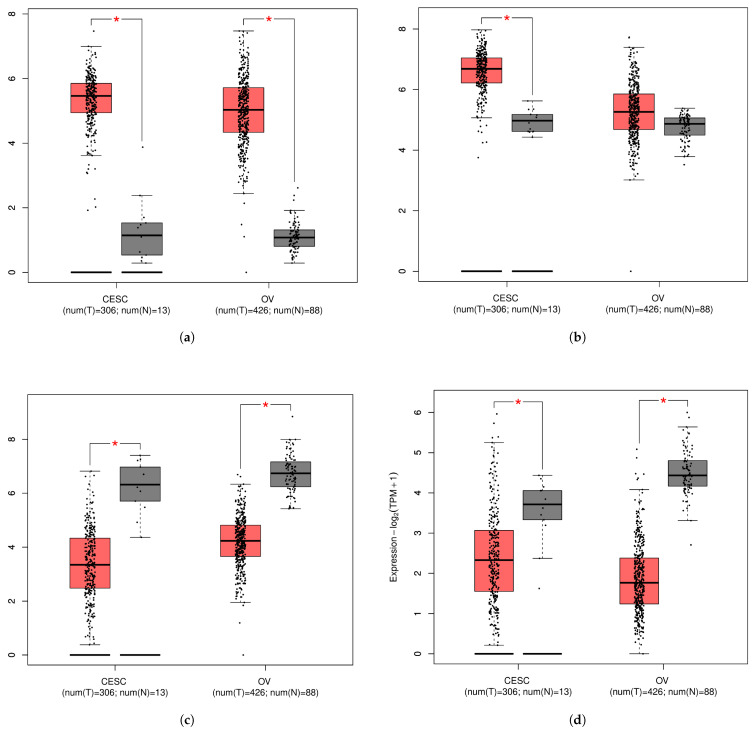
mRNA expression level analysis of HGs in CC and OC using TCGA data. Box plots illustrating the expression levels of (**a**) FOXM1, (**b**) MCM3, (**c**) SH3BP5, and (**d**) PAPSS2 in tumor (T) and normal (N) samples. Red indicates tumor samples, whereas black represents normal samples. The red asterisk (*) indicates a statistically significant difference between the two compared groups (Tumor vs. Normal).

**Figure 11:**
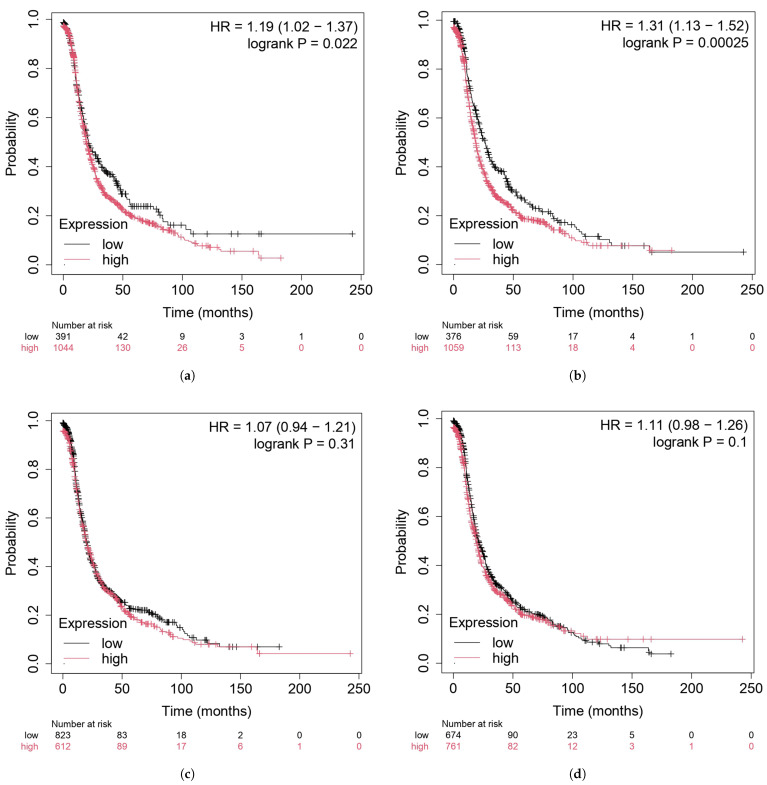
Survival analysis of HGs in CC using TCGA data; (**a**) FOXM1, (**b**) SH3BP5, (**c**) MCM3, and (**d**) PAPSS2. Red indicates the survival outcome for high expressions of the HGs, whereas black represents low expressions.

The expression levels of the identified HGs (FOXM1, MCM3, SH3BP5, and PAPSS2) were analyzed in cervical cancer and ovarian cancer using TCGA data. The box plots illustrate significant differential expression patterns between normal (N) and tumor (T) samples. FOXM1 (Figure 10a) and MCM3 (Figure 10b) were expressed at significantly higher levels in tumor tissues than in normal tissues, suggesting their potential involvement in tumor progression and proliferation. In contrast, SH3BP5 (Figure 10c) and PAPSS2 (Figure 10d) displayed significantly lower expression in tumor samples, indicating their possible roles as tumor suppressors. In the final phase of validation of the identified hub genes (HGs), survival analysis was employed. As illustrated in Figure 11, the identified HGs, particularly FOXM1 and SH3BP5, exhibited a significant association with poor survival outcomes.

### 3.7. Gene Regulatory Network Analysis

**Figure 12:**
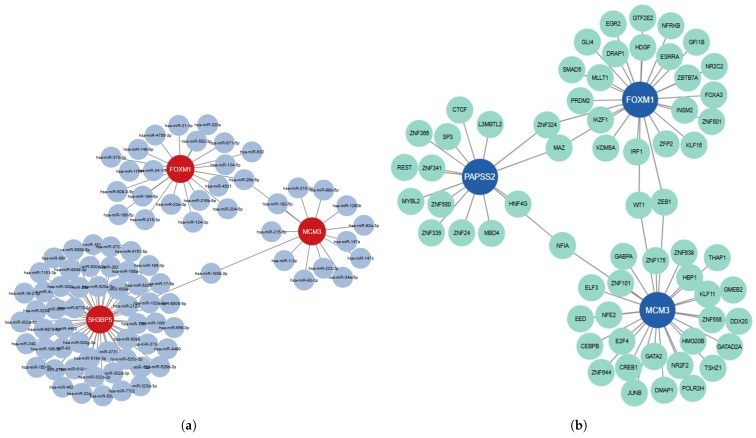
Gene regulatory networks of the identified HGs. (**a**) Gene–miRNA interaction network; (**b**) TFG interaction network.

GRN analysis of the identified HGs was conducted via the NetworkAnalyst. Figure 12 illustrates the gene–miRNA and TFG interaction networks of the identified HGs. The gene–miRNA interaction network (Figure 12a) consists of 90 nodes and 89 edges, depicting the regulatory correlations between key hub genes (FOXM1, SH3BP5, and MCM3) and their associated miRNAs. In this network, nodes in red color represent hub genes, blue nodes indicate miRNAs, and edges signify regulatory interactions, highlighting potential post-transcriptional regulation. The TFG interaction network (Figure 12b) comprises 66 nodes and 68 edges, showcasing interactions between hub transcription factors (FOXM1, PAPSS2, and MCM3) and their target genes. Here, blue nodes represent TFs, green nodes indicate target genes, and edges illustrate regulatory connections.

### 3.8. Molecular Docking Analysis

Molecular docking (MolDock) exploration was used to examine the interactions between the unveiled HGs and an FDA-approved drug for cervical and ovarian cancer: Olaparib. Binding affinities were evaluated via predefined thresholds, where binding energies below −4.25 kcal/mol suggest potential interactions, those below −5.0 kcal/mol represent moderate binding affinity, and values lower than −7.0 kcal/mol indicate strong binding interactions [47]. The detailed MolDock results are presented in Table 5. MolDock analysis revealed strong binding interactions between the identified HGs (FOXM1, MCM3, SH3BP5, and PAPSS2) and the FDA-approved drug. As shown in Table 5, all the binding energy values were below the threshold of −4.25 kcal/mol, indicating potential binding activity. Notably, Olaparib exhibited stronger binding affinities with all four target proteins, with the lowest binding energy observed for MCM3 (−11.2 kcal/mol), followed by FOXM1 (−9.5 kcal/mol). Similarly, PAPSS2 and SH3BP5 also significantly interact with Olaparib, with binding energies of −8.5 kcal/mol and −8.7 kcal/mol, respectively.

**Figure 13:**
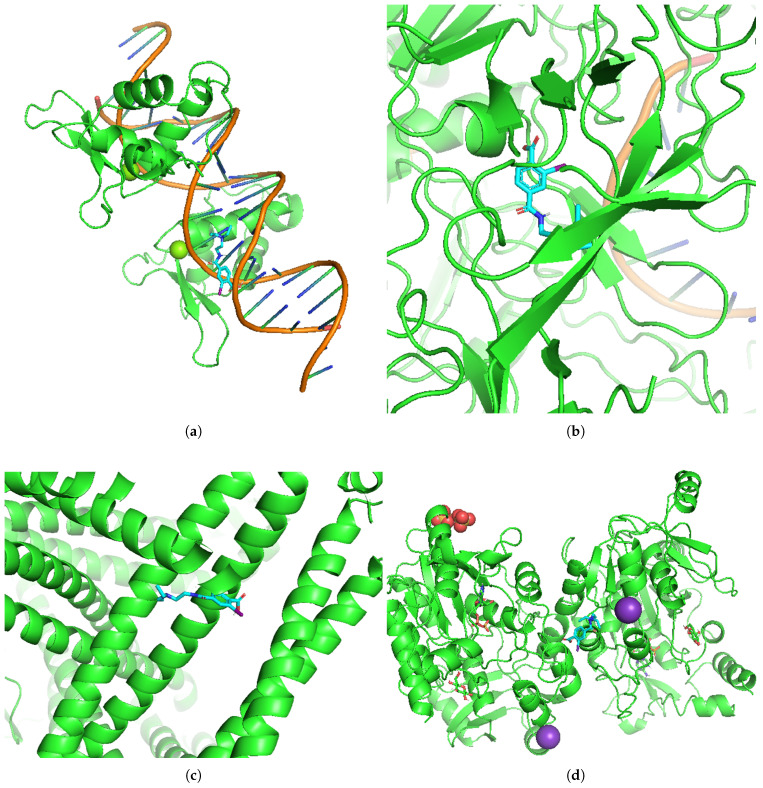
MolDock analysis of the identified HGs with the FDA-approved drug Olaparib. (**a**) FOXM1, (**b**) MCM3, (**c**) SH3BP5, and (**d**) PAPSS2. Proteins are shown in green, while ligands are represented in blue.

The results suggest that Olaparib has a greater binding affinity for the selected HGs, indicating its potential as a more effective therapeutic option for these proteins in cervical and ovarian cancer. The MolDock configurations are illustrated in Figure 13.

### 3.9. Discussion

Cervical and ovarian cancers are among the most lethal malignancies affecting the female reproductive system, underscoring the strong demand of pinpointing key biomarkers for early risk prediction and diagnosis. In this study, DEGs were uncovered from two microarray datasets, GSE63514 and GSE26712. Then, the two-step gene selection strategy employed in this study, integrating mRMR followed by SVM-RFE, proved highly effective in refining gene subsets that achieved superior classification accuracy across multiple models and datasets. This approach aligns well with the principles of Healthcare 4.0, which emphasizes precision, connectivity, and the intelligent use of big data for personalized medicine. Feature selection is especially critical in this framework because it mitigates the curse of dimensionality inherent in high-throughput genomic data, reducing redundancy and improving data interpretability. The mRMR method minimizes redundancy while maximizing gene relevance, preparing an optimal input for the subsequent SVM-RFE step, which further refines the subset by eliminating less informative features based on classification performance [12,17].

Classification plays a significant role in evaluating the discriminative power of the refined gene set [29]. In this regard, the attention mechanism can play a vital role to classify effectively. Attention mechanisms provide insight into which genes or features the model focuses on while making predictions. This interpretability is critical in biomedical applications to understand the biological relevance of key biomarkers [17,48,49]. By dynamically allocating weights to the most informative and relevant genes, attention helps distinguish subtle differences in gene expression patterns that may be pivotal for cancer prognosis and diagnosis. Moreover, the attention mechanism enhances model robustness by capturing complex, long-range dependencies within high-dimensional gene expression data, allowing for personalized feature interactions that differ among patients. This dynamic weighting not only improves predictive accuracy but also facilitates the identification of novel biomarkers by highlighting influential genes within the dataset [17,50], thereby aligning well with the goals of precision medicine in the Healthcare 4.0 paradigm. In our study, we used several classifiers, including XGBoost, AdaBoost, LightGBM, CNN, and AttLSTM, where AttLSTM outperformed other classifiers by effectively leveraging the attention mechanism to capture critical gene interactions and achieve superior discriminative power. To capture complex, nonlinear relationships among gene features, the AttLSTM network was utilized in our study, wherein the recurrent LSTM units learn contextual dependencies in the data and the attention mechanism prioritizes the most informative features during training, thereby enhancing the model’s representational capacity and classification performance. Using our proposed two-step feature selection method combined with an AttLSTM model, we identified a refined set of 1000 MDEGs.

Concurrently, WGCNA was implemented to identify coexpression-associated genes (CAGs). In the GSE63514 dataset, 16 modules were detected, with the yellow module containing 1248 CAGs being the most significant. Similarly, in the GSE26712 dataset, six modules were identified, among which the brown module, comprising 925 CAGs, showed the strongest association with the trait of interest. Enrichment analysis, including GO and KEGG pathway analyses, was conducted separately on the common MDEGs and CAGs. Both groups exhibited significant enrichment in various functional categories, illustrated in Figure 6 and Figure 7. Eight common genes were subsequently identified between all MDEGs and CAGs, as shown in Figure 8a.

A PPIN was then established via the NetworkAnalyst data repository and further visualized and analyzed in Cytoscape (Figure 8b). From this network, the top ten HGs were uncovered employing the degree centrality offered by the cytoHubba plugin, depicted in Figure 8c. Among these, genes with at least ten interactions were selected, resulting in the identification of four key HGs: FOXM1, MCM3, SH3BP5, and PAPSS2.

Forkhead Box M1 (FOXM1) is a critical regulator of the cell cycle and a member of the Forkhead transcription factor family, playing a vital role in embryonic development, cell proliferation, and cancer progression. As a proliferation-associated transcription factor, FOXM1 is present in all dividing cell types, including tumor cell lines, and has been closely linked to multiple solid tumors [51,52]. Its oncogenic properties allow it to influence a broad range of biological processes, emphasizing its significant contribution to tumor initiation and advancement [53]. It has been linked to several other cancers, including hepatocellular carcinoma (HCC) [53], small cell lung cancer [52], colorectal cancer [51], breast cancer [54], pancreatic cancer and esophageal cancer [55], and lung adenocarcinoma [56]. FOXM1 was also found to be overexpressed in cervical [57] and ovarian cancers [58], suggesting its involvement in their progression and development.

Minichromosome maintenance proteins (MCMs) are a group of six homologous proteins (MCM2-7) that play crucial roles in maintaining minichromosomes, initiating DNA replication, and regulating cell proliferation. Among them, MCM3 not only is cruicial for the initiation of DNA replication but also serves as a key marker for cellular proliferation [59,60]. MCM3 has been shown to be excessively expressed in cervical [61] and ovarian cancers [62], as well as in other malignancies such as HCC [60,63,64], NSCLC [59], and colorectal cancer [65]. Consistently, our study also revealed elevated expression of MCM3 in cervical and ovarian cancer samples compared to adjacent normal tissues. Both FOXM1 and MCM3 were significantly upregulated in cancer samples, whereas SH3BP5 and PAPSS2 were markedly downregulated in cancer samples compared with adjacent normal tissues. These HGs also achieved significant AUC scores in both the GSE63514 and GSE26712 datasets. Gene regulatory network (GRN) analysis provided critical insights into the regulatory mechanisms governing the identified hub genes. The gene–miRNA interaction network revealed key transcriptional regulatory elements, with FOXM1, SH3BP5, and MCM3 being significantly regulated by multiple miRNAs. Similarly, the TFG interaction network highlighted essential regulatory interactions, where hub TFs such as FOXM1, PAPSS2, and MCM3 were observed to control multiple target genes. Finally, these genes exhibited potential binding affinity with an FDA-approved drug compound, Olaparib. These observations imply that FOXM1, MCM3, SH3BP5, and PAPSS2 may serve as key biomarkers for early prognosis and diagnosis, as well as potential therapeutic targets for cervical and ovarian cancer.

## 4. Conclusions

In this study, key genes associated with cervical and ovarian cancer were pinpointed by integrating bioinformatics and machine learning approaches. Through differential expression analysis, ML-based feature selection, and WGCNA, we identified FOXM1, MCM3, SH3BP5, and PAPSS2 as potential biomarkers. These genes exhibited significant diagnostic potential, achieving high AUC scores during validation and displaying distinct expression patterns in cancer samples. Furthermore, drug–gene interaction analysis revealed their potential as therapeutic targets, demonstrating their binding affinity with an FDA-approved drug. Overall, these findings may enhance understanding of the molecular mechanisms driving cervical and ovarian cancer, highlighting promising biomarkers for early diagnosis and targeted therapy. Future research can utilize larger datasets and incorporate advanced machine learning techniques to further validate and refine these findings.

## Figures and Tables

**Table 1:** Comparison of the proposed method with related studies.

SN	Authors	Methods	Research Focus & Key Outcomes
1	Yang et al. [7]	Differential expression (DE) analysis and basic systems biology	Conducted biomarker identification using conventional bioinformatics approaches without ML-based methods.
2	Li et al. [19]	DE analysis with shallow ML algorithms, including RF, SVM-RFE, and LASSO	Identified hub genes from a set of DEGs and evaluated their diagnostic potential.
3	Zhou et al. [20]	LASSO, SVM, and RF	Identified common hub genes through feature selection without thoroughly evaluating discriminative power.
4	Jiao et al. [21]	scRNA-seq data analysis via LASSO regression	Revealed distinctive immune cell infiltration patterns and gene expression profiles within the tumor microenvironment (TME) of HGSOC.
5	**Proposed method**	Two-step gene selection with discriminative efficacy evaluation using AttLSTM; WGCNA	Removed redundant genes with similar expression patterns and applied an attention mechanism to capture complex, nonlinear relationships among genes.

**Table 2:** Description of the datasets.

Dataset	Platform	Tumor	Normal	Total Samples
GSE63514	GPL570	46	24	195
GSE26712	GPL96	104	24	128

**Table 3:** Classification performance comparison of various models on gene sets selected by mRMR and SVM-RFE.

Dataset	Gene Set	Method	ACC	AUC	PRE	REC	F1
GSE63514	mRMR+SVM-RFE	XGBoost	88.46±0.71	0.975±0.006	1.000±0.000	0.869±0.021	0.930±0.012
		AdaBoost	88.46±0.83	0.961±0.008	0.666±0.031	0.800±0.025	0.727±0.019
		LightGBM	92.31±0.65	0.962±0.005	0.901±0.018	1.000±0.000	0.953±0.010
		CNN	84.37±0.92	0.903±0.009	1.000±0.000	0.843±0.022	0.915±0.014
		AttLSTM	96.15±0.42	0.990±0.003	0.952±0.009	1.000±0.000	0.975±0.006
GSE26712	mRMR+SVM-RFE	XGBoost	97.43±0.48	1.000±0.000	1.000±0.000	0.972±0.011	0.985±0.007
		AdaBoost	97.44±0.51	0.986±0.004	0.666±0.030	1.000±0.000	0.800±0.020
		LightGBM	97.96±0.39	1.000±0.000	0.833±0.017	0.988±0.008	0.981±0.006
		CNN	97.43±0.44	1.000±0.000	1.000±0.000	0.974±0.010	0.987±0.005
		AttLSTM	99.28±0.31	1.000±0.000	1.000±0.000	1.000±0.000	0.998±0.002

**Table 4:** Comparative performance analysis of mRMR+SVM-RFE against other single-step feature selection techniques.

Dataset	Feature Selection	ACC	AUC	PRE	REC	F1
GSE63514	Extra Trees	92.30±0.68	0.942±0.007	0.852±0.021	0.866±0.020	0.749±0.018
	MiG	90.42±0.74	0.933±0.009	0.952±0.015	0.936±0.019	0.884±0.013
	ANOVA	94.54±0.56	0.933±0.006	0.912±0.017	0.900±0.018	0.895±0.010
	LASSO	93.76±0.61	0.944±0.005	0.952±0.013	0.915±0.016	0.927±0.009
	**mRMR+SVM-RFE**	96.15±0.42	0.990±0.003	0.952±0.009	1.000±0.000	0.975±0.006
GSE26712	Extra Trees	94.87±0.52	0.983±0.004	1.000±0.000	0.948±0.012	0.973±0.007
	MiG	95.56±0.47	0.978±0.006	0.988±0.010	0.959±0.014	0.975±0.006
	ANOVA	97.48±0.41	1.000±0.000	1.000±0.000	0.977±0.009	0.973±0.006
	LASSO	96.72±0.45	0.994±0.003	0.987±0.011	0.958±0.013	0.984±0.005
	**mRMR+SVM-RFE**	99.28±0.31	1.000±0.000	1.000±0.000	1.000±0.000	0.998±0.002

**Table 5:** Molecular docking-based interaction analysis.

Gene Name	Drug Compound	Docking Score (kcal/mol)
FOXM1	Olaparib	−9.5
MCM3	Olaparib	−11.2
SH3BP5	Olaparib	−8.7
PAPSS2	Olaparib	−8.5

## Data Availability

The datasets are publicly available in the Gene Expression Omnibus (GEO) repository (https://www.ncbi.nlm.nih.gov/geo/, accessed on 24 March 2025).

## Abbreviations

The following abbreviations are used in this manuscript:

DEGs	Differentially Expressed Genes
MDEGs	ML-based DEGs
WGCNA	Weighted Gene Coexpression Network
PPIN	Protein–Protein Interaction Network
GRN	Gene Regulatory Network
MolDock	Molecular Docking
AttLSTM	Attention-based LSTM
